# Medical students find assessments stressful. Of course….but what do we do about it?

**DOI:** 10.1007/s40037-014-0152-x

**Published:** 2014-12-10

**Authors:** Vicki R. LeBlanc

**Affiliations:** 1 Wilson Centre, Toronto, Canada; 2 Faculty of Dentistry and Department of Medicine, University Health Network and University of Toronto, 200 Elizabeth St, 1ES-565, Toronto, ON M5G 2C4 Canada

In this issue, Lyndon et al. [1] present the results of a systematic review on the relationship between academic assessment and psychological distress among medical students. One of their main findings is that different types of assessments are associated with subjective and physiological stress in medical students. As someone who has studied emotions, particularly stress, for 10 years, it would be easy for my response to be ‘of course!’ Medical training and practice are awash with emotional situations, from undergoing high stakes assessments, to the fear of making costly mistakes, to witnessing heartbreaking loss and death. However, this aspect of medical education has, for the most part, been ignored or merely superficially addressed in our study of how medical students learn and perform [2]. The Lyndon et al. paper [1] shines a light on an important and often neglected aspect of learning and performance.

Research on how medical trainees learn, reason through clinical situations, or interact with patients has tended to focus on the knowledge and skills they require and how to develop them. However, there is overwhelming evidence from the psychological and neuroscience domains showing that emotions cannot be separated from cognitive functions [3]. We, as humans, are always in an emotional state of some kind. These emotions are instrumental in shaping how we attend to the world, what we remember from our experiences and how we make decisions. While there is growing interest in this topic in medical education, Lyndon et al. [1] highlight the nascent nature and disparity of the research to date on this topic. They are left, as is often the case for meta-analyses in medical education, with one sure conclusion: more research is needed.

In our continued study of the impact of emotions, it is essential that research be guided by current theories, and that it builds on what is already known beyond medical education. As a construct, stress is highly complex. As an experience, however, it is powerfully familiar. As a result, we have many common sense beliefs about emotions. Furthermore, outdated theories, such as the Yerkes–Dodson law discussed by Lyndon et al. [1], maintain their appeal because of their simplicity and because they feel right. However, these common sense beliefs and theories do not capture the true complexity of the emotional response and the mechanisms by which they influence learning and performance. In contrast, current models of emotions, based on appraisal processes [3, 4], provide an elegant mechanism to explain stress responses: When faced with a situation (e.g. in-training examination) that can impede the achievement of a closely held goal (being top of class, becoming a doctor), a person makes an appraisal of the demands of the situation (how challenging is the exam) and of their resources to meet those demands (how much do I know? how well can I perform the skill?). If the resources are thought to meet the demands, then a positive state of eustress occurs and the individual mobilizes energy to the task (productive preparation). If, however, the demands are thought to outweigh the resources (I don’t know the Krebs cycle) or there is uncertainty (I don’t know what this instructor is looking for in my performance), then the person will feel distress. Thus, current models of stress, rather than fitting the curvilinear pattern of the Yerkes–Dodson law, resemble a more qualitative shift. This state of distress, as has been shown by a wealth of research, is accompanied by the activation of certain physiological systems (leading to the release of cortisol and alpha-amylase), and subjective experiences of fear, anxiety, agitation, etc. Both the physiological and the subjective responses influence attention, memory and decision-making processes [3]. If we are to truly understand the mechanisms of stress, and its impact on learning and performance, future research endeavours need to build on what is already known beyond the field of medical education.

In addition to the need for more rigorous research on emotions and their impact on learning and performance, medical educators are tasked with a practical challenge. How do we deal with the fact that students are stressed by their academic and clinical environments? One approach could be to do nothing, to leave the onus on the individual to develop the necessary coping skills needed to become a health professional. The problem with taking this approach, which has essentially been our *modus operandus* for the past 30 years, is that not everyone will develop adaptive coping styles. In the health professions, the rates of posttraumatic stress, burnout, sick leave and absenteeism are high [5, 6]. Left to their own devices, many health professionals develop maladaptive ways to cope with the stressors of training and practice, to the detriment of their mental health and performance.

An alternative is to select, in our admissions processes, those individuals who can handle the emotional load of medicine. An example of this approach is the recent interest in screening incoming medical students for their emotional intelligence [7]. There are several challenges with this approach, however [8]. The first is that constructs like emotional intelligence are currently of little value to educators. They are too general and they mean too many things to too many people. A second challenge is that we do not know the characteristics of those individuals who are resilient to the stressors of medical training and practice. Stress is a very situation-specific response. A person can get stressed by a verbal exam (due to being in the ‘hot seat’ or the ‘fish bowl’) and yet think nothing of a trauma resuscitation that would be quite gruesome to the average person. There do not appear to be a general set of stable characteristics that predict who will cope well with the emotional world of medicine.

We can’t continue to neglect the emotional aspects of medical training, and it would be unethical to screen for resilience, because we don’t know what factors predict resilience to the stressors of medical training and practice. What we can—and must—do, as educators, is provide students with the skills required to maintain cognitive function during emotional events. This goal is achievable. Effective methods of emotional regulation, such as stress inoculation training and approaches that target situation reappraisal rather than emotional suppression, can reduce subjective and physiological stress and increase cognitive function and performance [9–12]. This is a promising area of future research for medical education.

In summary, Lyndon et al. [1] highlight an important, and often neglected, area of medical education. They also show that research into the emotional aspects of medical training is sparse and the findings disparate. My hope is that their paper will serve to foster more research, building on what is already known outside of medicine. The ultimate goal is to better understand the emotional aspect of medical training and, as a result, to better prepare future health professionals.
